# Trajectories of occupational physical activity and risk of later-life mild cognitive impairment and dementia: the HUNT4 70+ study

**DOI:** 10.1016/j.lanepe.2023.100721

**Published:** 2023-08-29

**Authors:** Ekaterina Zotcheva, Bernt Bratsberg, Bjørn Heine Strand, Astanand Jugessur, Bo Lars Engdahl, Catherine Bowen, Geir Selbæk, Hans-Peter Kohler, Jennifer R. Harris, Jordan Weiss, Sarah E. Tom, Steinar Krokstad, Teferi Mekonnen, Trine Holt Edwin, Yaakov Stern, Asta Kristine Håberg, Vegard Skirbekk

**Affiliations:** a Department for Physical Health and Aging, Norwegian Institute of Public Health, Oslo, Norway; b Norwegian National Centre of Ageing and Health, Vestfold Hospital Trust, Tønsberg, Norway; c Centre for Fertility and Health, Norwegian Institute of Public Health, Oslo, Norway; d Ragnar Frisch Center for Economic Research, Oslo, Norway; e Department of Geriatric Medicine, Oslo University Hospital, Oslo, Norway; f Department of Global Public Health and Primary Care, University of Bergen, Bergen, Norway; g Independent Researcher, Vienna, Austria; h Faculty of Medicine, University of Oslo, Oslo, Norway; i Population Aging Research Center and Department of Sociology, University of Pennsylvania, Philadelphia, PA, USA; j Stanford Center on Longevity, Stanford University, Stanford, CA, USA; k Department of Neurology, Columbia University Vagelos College of Physicians and Surgeons, USA; l Department of Epidemiology, Columbia University, Mailman School of Public Health, USA; m HUNT Research Centre, Department of Public Health and Nursing, Faculty of Medicine and Health Sciences, Norwegian University of Science and Technology, Trondheim, Norway; n Levanger Hospital, Nord-Trøndelag Hospital Trust, Norway; o Department of Neuromedicine and Movement Science, Faculty of Medicine and Health Sciences, Norwegian University of Science and Technology, Trondheim, Norway

**Keywords:** Aging, Dementia, Minor cognitive impairment, Occupational physical activity

## Abstract

**Background:**

High levels of occupational physical activity (PA) have been linked to an increased risk of dementia. We assessed the association of trajectories of occupational PA at ages 33–65 with risk of dementia and mild cognitive impairment (MCI) at ages 70+.

**Methods:**

We included 7005 participants (49.8% were women, 3488/7005) from the HUNT4 70+ Study. Group-based trajectory modelling was used to identify four trajectories of occupational PA based on national registry data from 1960 to 2014: stable low (30.9%, 2162/7005), increasing then decreasing (8.9%, 625/7005), stable intermediate (25.1%, 1755/7005), and stable high (35.2%, 2463/7005). Dementia and MCI were clinically assessed in 2017–2019. We performed adjusted multinomial regression to estimate relative risk ratios (RRR) with 95% confidence intervals (CI) for dementia and MCI.

**Findings:**

902 participants were diagnosed with dementia and 2407 were diagnosed with MCI. Absolute unadjusted risks for dementia and MCI were 8.8% (95% CI: 7.6–10.0) and 27.4% (25.5–29.3), respectively, for those with a stable low PA trajectory, 8.2% (6.0–10.4) and 33.3% (29.6–37.0) for those with increasing, then decreasing PA; while they were 16.0% (14.3–17.7) and 35% (32.8–37.2) for those with stable intermediate, and 15.4% (14.0–16.8) and 40.2% (38.3–42.1) for those with stable high PA trajectories. In the adjusted model, participants with a stable high trajectory had a higher risk of dementia (RRR 1.34, 1.04–1.73) and MCI (1.80, 1.54–2.11), whereas participants with a stable intermediate trajectory had a higher risk of MCI (1.36, 1.15–1.61) compared to the stable low trajectory. While not statistically significant, participants with increasing then decreasing occupational PA had a 24% lower risk of dementia and 18% higher risk of MCI than the stable low PA group.

**Interpretation:**

Consistently working in an occupation with intermediate or high occupational PA was linked to an increased risk of cognitive impairment, indicating the importance of developing strategies for individuals in physically demanding occupations to prevent cognitive impairment.

**Funding:**

This work was supported by the 10.13039/100000002National Institutes of Health (R01AG069109-01) and the 10.13039/501100005416Research Council of Norway (296297, 262700, 288083).

**Research in context**

**Evidence before this study**

We searched PubMed and individual article reference lists to find relevant studies from December 15th 2022 to April 1st 2023. The search terms included, but were not limited to, *occupation∗*, *physical activity, characteristics, dementia, mild cognitive impairment, cognitive impairment, work∗, physical demands,* and *physically demanding*. Existing evidence suggests that individuals working in occupations with high physical activity are at an increased risk of cognitive impairment later in life, compared to individuals working in less physically demanding occupations.

**Added value of this study**

Our findings extend those from previous studies by incorporating a life-course perspective into research on occupational physical activity and cognitive impairment. Whereas previous studies have mainly focused on a single measurement of occupation, often close to retirement, we include occupational trajectories from ages 33–65 to give a broader picture of the occupational histories of the participants and how these relate to risk of cognitive impairment in later adulthood. We also show that the increased risk of dementia and MCI in individuals with high occupational physical activity persists after accounting for factors such as education and income. In addition, our use of rich national registry data on occupation provides additional strength to our findings by reducing the biases commonly associated with self-report.

**Implications of all the available evidence**

The observed associations of high occupational physical activity and increased risk of late-life cognitive impairment indicates that special attention must be given to workers in these occupations, warranting strategies aimed at preventing or reducing cognitive impairment.

## Introduction

With a rapidly growing population of older adults worldwide, identifying modifiable risk factors for cognitive decline and impairment is receiving increasing attention. While there has been a focus on educational attainment, the role of other modifiable factors in the prevention of age-related cognitive decline and impairment has received less attention. However, accumulating evidence points towards the influence of occupational characteristics on cognitive health (for a review, see Burzynska et al.^1^).

Although higher levels of leisure-time physical activity (PA) are robustly related to lower risk of cognitive impairment,^2^, ^3^, ^4^ high physical demands *in the workplace* have been linked to higher dementia risk.^5^, ^6^, ^7^ For instance, Nabe-Nielsen et al.^5^ found that men who reported high occupational PA when they were 40–59 years old had a 48% higher dementia risk at ages 60+ than individuals with predominantly sedentary jobs. Similarly, Rovio et al.^6^ found an increased dementia risk after a mean follow-up of 21 years in individuals with higher occupational PA, although those estimates had wide confidence intervals and did not reach statistical significance. A case–control study found that higher occupational physical demands when the participants were in their 20s, 40s, and 50s were associated with an increased odds of Alzheimer's disease later in life.^7^ The inverse associations between leisure-time and occupational PA with health-related outcomes has been termed the “PA paradox”,^8^, ^9^, ^10^, ^11^, ^12^ although substantial confounding by socioeconomic factors has been suggested.^13^ As a result, the recent World Health Organization guidelines for PA and sedentary behavior take the PA paradox into consideration and call for further research on the effects of occupational and leisure-time PA on health.^14^ High occupational PA (and especially excessive relative aerobic workloads experienced by aging workers) could potentially raise the risk of dementia through cardiovascular stress.^15^

Prior studies on occupational PA and dementia have several limitations. Occupational PA has mainly been self-reported, i.e., participants were asked to recall and interpret the extent to which their job was physically demanding.^5^^,^^6^ However, self-reported physical activity can have low external validity^16^ and correlates poorly with objectively measured assessments.^17^ Further, self-reports are prone to recall bias and misinterpretation, especially in older populations where recall and interpretation may be affected by changes in cognitive function.^18^ In addition, prior studies have typically assessed occupation at a single time-point in the individual's career.^5^^,^^6^ Because individuals can change jobs and/or occupations over the course of their working lives, such single-point assessments may be misleading, especially when assessments take place close to dementia onset. Since the preclinical period of dementia may start up to two decades prior to symptom onset,^19^ a life-course approach where different occupations during the working life are taken into account could provide more accurate information on the complex relationships between occupational characteristics and cognitive impairment.

In Norway, a resident's occupational history is prospectively registered and coded according to the International Standard Classification of Occupations-88 (ISCO) by Statistics Norway.^20^ This makes it possible to link individual occupational data to various indices of occupational characteristics, such as the Occupational Information Network (O^∗^NET).^21^ O^∗^NET is a comprehensive database of the characteristics of over 900 occupations based on data from employees, occupational experts, and occupational analysts. This provides a unique possibility to use standardized as opposed to self-reported data to investigate the relationship between occupational PA and later-life cognitive impairment following a life-course perspective.

Utilizing registry or administrative job task and occupational exposure matrix approaches can represent an important avenue for research into chronic disease outcomes with long latency periods or where cumulative repetitive exposures matter.^22^ The aim of the present study was to examine how registry-based trajectories of occupational PA at age 33–65 years are related to the risk of dementia and mild cognitive impairment (MCI) at ages 70+ years. In line with previous research,^5^, ^6^, ^7^ we hypothesized that individuals who consistently work in occupations with low levels of occupational PA would have the lowest risk of dementia and MCI compared to individuals who consistently work in occupations with high levels of occupational PA.

## Methods

### Study population

**Fig. 1:**
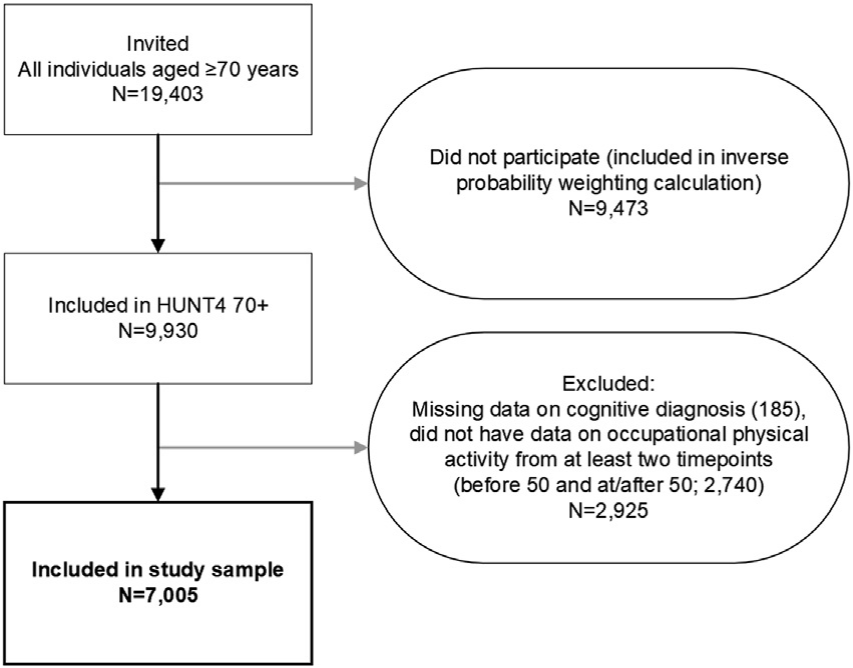
Study sampling scheme.

We used a historical cohort design in which cognitive outcomes at age 70+ years were linked to trajectories of occupational PA at age 33–65 years. The study sample comprised 7005 participants from the 70+ sub-study of the fourth wave of the Trøndelag Health Study in Norway (HUNT4 70+, 2017–2019). The HUNT Study is a large ongoing population-based health survey performed in the Trøndelag County in Norway, where all adult residents were invited to participate in four surveys: HUNT1 (1984–1986), HUNT2 (1995–1997), HUNT3 (2006–2008), and HUNT4 (2017–2019). In the HUNT4 Survey, all county inhabitants aged 70 years and older (n = 19,403) were invited to participate in the 70+ sub-study, and 9930 (51.2%) individuals aged 70–105 participated. In the present study, we included 7005 (72.0%) participants aged 70–105 (born in 1914–1949) from the HUNT4 70+ study for whom data from the cognitive assessment and data on occupational PA from at least two time points were available; notably, at least once before the age of 50 years, and at least once at or after the age of 50 years (Fig. 1). This restriction was set to exclude participants who had only worked early or later in life. As there were insufficient occupational data at the ages of 30–32 years to create trajectories starting at 30 years, the trajectories included occupational PA from ages 33–65 years. The upper cut-off of 65 years was set based on the mean age at first retirement pension withdrawal in Norway as of 2022 (65.6 years).^23^ All participants provided informed, written consent. See Figure S1 for an overview of the modelling approach using a directed acyclic graph (DAG).

### Occupational physical activity

Individual data on occupation were obtained from administrative registers with full population coverage from Statistics Norway and linked to data on our study participants using the unique personal identification number assigned to all registered residents of Norway. Annual data were available for 1960, 1970, 1980, and the years spanning 1995–2014. The main occupation for each participant in each year was defined as the occupation which yielded the highest salary that year. Occupation was coded using four-digit occupation codes according to the Norwegian version of the ISCO-88 Standard.^20^ Our data included workers from 305 occupations. For 1960, 1970, and 1980, we obtained occupational codes by using a crosswalk to impute ISCO-88 codes based on codes from the Nordic Standard of Occupations.^24^ For 1995–2002, there were no data on occupation in the employer-employee registry. For these years, the occupational codes were imputed by using a crosswalk based on combinations of a four-digit industry of employment code^25^ and a four-digit educational code.^26^ The crosswalk was constructed using years with complete occupation, industry, and education data to assign the mode occupation to each industry-by-education cell in the full Norwegian labor force.

To determine occupational PA, the ISCO-88 occupational codes were linked to data from O^∗^NET (2003 version)^21^ to establish the degree to which each occupation involved performing general PA: “4.A.3.a.1 Performing General Physical Activities: Performing physical activities that require considerable use of your arms and legs and moving your whole body, such as climbing, lifting, balancing, walking, stooping, and handling of materials”.^21^ The level of occupational PA was ranked on a scale of 1–5, with higher scores indicating higher occupational PA. In our study sample, the mean occupational PA level was 3.06 (SD 0.93). The resulting scores were standardized, which translates to a change of 1 SD on the standardized score being equal to a shift of 0.93 points on the occupational PA index (the original data scale).

### Dementia and MCI

All participants in HUNT4 70+ were clinically evaluated for dementia and MCI using a comprehensive assessment of cognitive function, activities of daily living, neuropsychiatric symptoms, disease course, and a family caregiver interview.^27^ Each participant was independently assessed by two out of nine specialists in geriatrics, neurology, or old age psychiatry, according to the DSM-5 diagnostic criteria,^28^ and categorized as no cognitive impairment, MCI (mild neurocognitive disorder), or dementia (major neurocognitive disorder). For more details on the diagnostic workup in HUNT4 70+, see Gjøra et al.^27^ An MCI diagnosis is not necessarily followed by dementia. The outcomes were hence considered not strictly ordinal, and multinomial logistic regression was used.^29^

### Other covariates

Data on age, sex, education (highest achieved level of education: primary, secondary, or tertiary), marital status (from 1986, corresponding to time at HUNT1: unmarried, married, widow, divorced/separated), retirement age (either regular age retirement, early retirement, or disability retirement), and income (mean annual income in EUR at ages 45–55, inflated to 2018 currency and grouped into quintiles) were obtained from national registries. Annual income commonly peaks around age 45–55. It is also less influenced by childbearing and breaks due to higher education found in earlier ages and avoids early retirement common in some occupations.^30^ The remaining variables were obtained from questionnaires and measurements at HUNT1 (1984–1986) and HUNT2 (1995–1997). Data from HUNT3 (2006–2008) were not included in our analyses, as the data collection at HUNT3 occurred after retirement for many of the study participants, meaning that measurements at HUNT3 were highly unlikely to be confounders in the associations between trajectories of occupational PA and cognitive impairment. Participants were classified as hypertensive if their blood pressure measurements corresponded to hypertension (≥140 mmHg systolic blood pressure and/or ≥90 diastolic blood pressure based on the mean of two measurements at HUNT1 and the mean of three measurements at HUNT2) at HUNT1 and/or HUNT2. Cardiovascular disease (CVD) was categorized into no/yes, according to whether participants self-reported ever having myocardial infarction, angina pectoris, or stroke at HUNT1 and/or HUNT2. Psychiatric and somatic impairment were categorized into no/yes, according to whether participants self-reported having a moderate or severe psychiatric or somatic impairment at HUNT1 and/or HUNT2 (the surveys did not ask for diagnosis). Participants were classified as obese if their body mass index (BMI) was ≥30 kg/m^2^ at HUNT1 and/or HUNT2.^31^ Insufficient leisure-time PA (performing less than 30 minutes of PA per day^32^) was categorized into no/yes, according to participants’ self-reported PA at HUNT1 and/or HUNT2.

### Statistical analyses

Group-based trajectory modelling using the traj package in Stata 17.0^33^ was conducted to identify groups of study participants who shared similar underlying trajectories of occupational PA between 33 and 65 years of age (see Supplemental Methods for details). Each participant needed to have a minimum of two occupational data points: one or more before the age of 50, and one or more at or after the age of 50. Tables S1–S5 and S6, and Figure S2 in the Appendix provide further details on characteristics for the trajectory modeling, as well as measures of model fit and examples of the most common occupations in each of the trajectory groups.

**Fig. 2:**
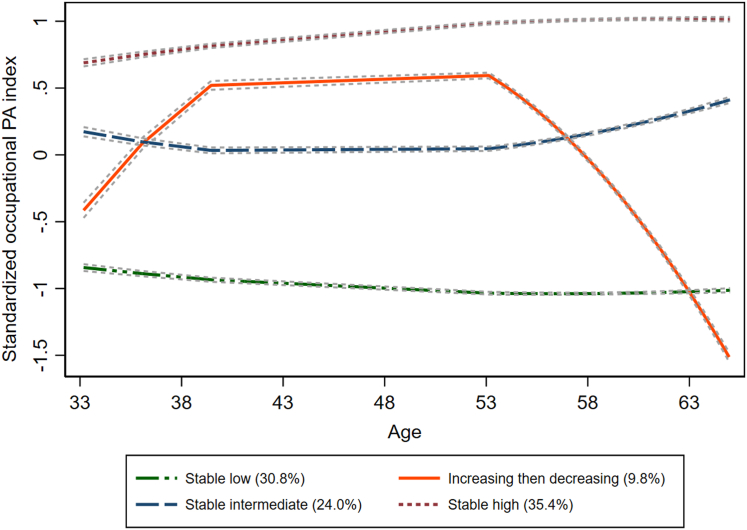
Trajectory plot of standardized occupational physical activity (PA) at ages 33–65 years showing four distinct trajectory groups: (1) stable low occupational PA, (2) increasing then decreasing occupational PA, (3) stable intermediate occupational PA, and (4) stable high occupational PA. A standardized occupational PA score of zero is equivalent to a score of 3.06 on the occupational PA index in O^∗^NET. One SD is equivalent to a 0.93 point increase or decrease on the unstandardized occupational PA index. The dashed grey lines surrounding each trajectory are 95% confidence intervals on the estimated probabilities of group membership. Percentages in the legend represent the estimated group probability. For data on each participant's trajectory per occupational PA group, see Figure S2, and for the distribution of the occupational PA index in the sample, see Figure S3. Typical occupations for the occupational PA groups can be found in Tables S5 and S6.

The decision on the number and shape of the trajectory groups was based on standard criteria^34^^,^^35^; the Bayesian Information Criterion (BIC) indicating goodness-of-fit of the models, the posterior probability indicating internal reliability, the odds of correct classification (≥5), and whether the model reflected the data in a comprehensible and analytically tractable manner (see Tables S1 and S2). Fig. 2 illustrates the final trajectory model consisting of four groups: *stable low* (estimated group probability: 30.8%; proportion assigned to group: 30.9%), *increasing then decreasing* (estimated group probability: 9.8%; proportion assigned to group: 8.9%)*, stable intermediate* (estimated group probability: 24.0%; proportion assigned to group: 25.1%), and *stable high* (estimated group probability: 35.4%; proportion assigned to group: 35.2%) that were used in the final statistical analyses. Figure S2 shows individual trajectories of the standardized occupational PA index by age for the study participants, stratified by trajectory group.

**Fig. 3:**
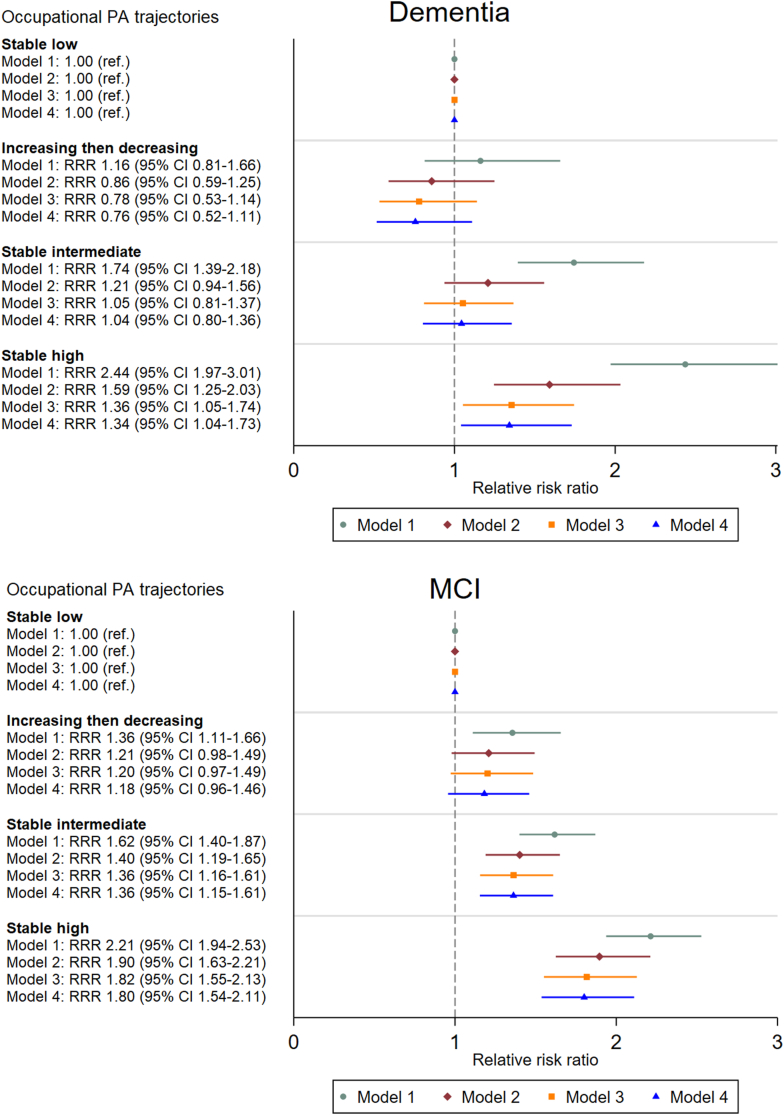
Relative risk ratios with 95% confidence intervals for the associations of occupational PA trajectories at ages 33–65 with risk of dementia and MCI in the HUNT4 70+ Survey (n = 7005). Model 1: age- and sex-adjusted; Model 2: education was added to Model 1; Model 3: mean annual income at ages 45–55 was added to Model 2; Model 4: marital status, hypertension, CVD, psychiatric impairment, somatic impairment, insufficient leisure-time PA, and obesity were added to Model 3.

We used inverse-probability weighting (IPW) to account for non-response in HUNT4 70+ and multiple imputation (MI) with 20 iterations to impute missing values on potential covariates. Details on the IPW and MI procedures are provided in the Supplemental Methods in the Appendix. Multinomial logistic regression with “no cognitive diagnosis” as the base outcome was used to estimate relative risk ratios (RRRs) with 95% confidence intervals (CI) for dementia and MCI associated with the four different occupational PA trajectories, see Fig. 3. Although MCI is often considered an intermediate stage between normal cognition and dementia, according to a meta-analysis most people with MCI do not progress to dementia even after 10 years of follow-up.^36^^,^^37^ We therefore used multinomial as opposed to ordinal logistic regression. The stable low occupational PA trajectory served as the reference category in the analyses. Adjustment variables were selected based on an a priori created DAG (Figure S1). The four regression models successively adjusted for age and sex (Model 1), education (Model 2), income (Model 3), and marital status, CVD, psychiatric or somatic impairment, hypertension, obesity, and insufficient leisure-time PA (Model 4). Interactions of trajectories of occupational PA with age, sex, and education were investigated by adding interaction terms to the regression model. Finally, we performed sensitivity analyses on complete case data (n = 5940, 84.8%) without IPW.

### Role of the funding source

The funders of the study had no role in study design, data collection, data analysis, data interpretation, or writing of the report. The corresponding author had full access to all the data in the study and had final responsibility for the decision to submit for publication.

## Results

Table 1 shows descriptive characteristics of the study sample (n = 7005, 49.8% women, and a mean age of 77.3 [SD 6.1] years in 2018). In the sample, 902 participants were diagnosed with dementia and 2407 were diagnosed with MCI. Absolute unadjusted risks for dementia and MCI were 8.8% (95% CI 7.6–10.0) and 27.4% (25.5–29.3), respectively, for those with a stable low PA trajectory, 8.2% (6.0–10.4) and 33.3% (29.6–37.0) for those with increasing, then decreasing PA; while it was 16% (14.3–17.7) and 35% (32.8–37.2) for those with stable intermediate, and 15.4% (14.0–16.8) and 40.2% (38.3–42.1) for those with stable high PA trajectories.

**Table 1:** Study sample characteristics of 7005 participants from the HUNT4 70+ Study born 1914–1949 grouped by trajectories of occupational physical activity (PA).

	Stable low (n = 2162)	Increasing, then decreasing (n = 625)	Stable intermediate (n = 1755)	Stable high (n = 2463)
**Registry-based**				
Age in 2018, mean (SD)	76.5 (5.6)	75.6 (5.3)	78.7 (6.7)	77.5 (6.1)
Sex (women)	958 (44.3)	256 (41.0)	810 (46.2)	1466 (59.5)
Education				
Primary	73 (3.4)	103 (16.5)	468 (26.7)	785 (31.9)
Secondary	774 (35.8)	437 (69.9)	1131 (64.4)	1439 (58.4)
Tertiary	1315 (60.8)	85 (13.6)	156 (8.9)	239 (9.7)
Married	1917 (88.7)	544 (87.0)	1548 (88.2)	2126 (86.3)
Retirement age, mean (SD)	66.0 (3.5)	65.9 (3.8)	65.3 (4.3)	64.7 (4.5)
Annual income age 45–55 years, EUR, mean (SD)	66,015 (24,148)	57,808 (27,253)	49,280 (26,876)	43,688 (19,191)
**Occupational PA**				
Standardized occupational PA index, mean (SD)				
Age 30–39	−0.88 (0.68)	−0.29 (0.96)	0.16 (0.72)	0.74 (0.67)
Age 40–49	−0.98 (0.54)	0.69 (0.66)	−0.01 (0.67)	0.84 (0.61)
Age 50–59	−1.05 (0.44)	0.66 (0.67)	0.08 (0.57)	0.98 (0.44)
Age 60–69	−1.04 (0.41)	−0.78 (0.70)	0.29 (0.55)	1.03 (0.41)
**HUNT1-HUNT2**				
Insufficient leisure-time PA	655 (30.3)	260 (41.6)	690 (39.3)	1125 (45.7)
Obesity	227 (10.5)	87 (13.9)	244 (13.9)	414 (16.8)
Hypertension	931 (43.1)	297 (47.5)	911 (51.2)	1221 (49.6)
CVD	60 (2.8)	20 (3.2)	81 (4.6)	93 (3.8)
Psychiatric impairment	44 (2.0)	17 (2.7)	42 (2.4)	53 (2.2)
Somatic impairment	118 (5.5)	39 (6.2)	161 (9.2)	216 (8.8)
**HUNT4 70+**				
Dementia	191 (8.8)	51 (8.2)	281 (16.0)	378 (15.4)
MCI	593 (27.4)	208 (33.3)	615 (35.0)	991 (40.2)

The data shown here are not imputed.

Numbers are n (%) unless stated otherwise.

CVD: cardiovascular disease; HUNT: Trøndelag Health Study; HUNT1: 1985–1986; HUNT2: 1995–1997; HUNT4 70+: 2017–2019; MCI: mild cognitive impairment. See Fig. 2 for the occupational PA trajectory plots, Table S4 for information on not-included and non-participants’ sociodemographic and occupational physical activity charateristics, and Figure S3 for the distribution of occupational PA index in the study sample versus the non-participants in HUNT4 70+ survey.

Table S4 displays sociodemographic and occupational characteristics of the HUNT4 70+ sample included in this study, of those with insufficient data to calculate occupational PA trajectories in HUNT4 70+ and hence not included in the study sample, and of those invited who chose not to participate (non-participants in HUNT4 70+). The sample included in our study was better educated, had a higher income, and were healthier than their peers who were not included or chose not to participate. The study sample covers the range of occupational PA in the underlying population, as shown in Figure S3 which portrays the distribution of the occupational PA index in the study sample and among non-participants in the HUNT4 70+ survey. As the figure reveals, there is a similar distribution of occupational PA in the two samples, the main exception being a higher share in the study sample with low-to-intermediate occupational PA (e.g., teachers) as well as a somewhat lower share in the high occupational PA range (e.g., construction workers). Taken together, the study sample is somewhat positively selected in terms of health and education, income, and lower occupational PA.

Table S5 shows the most frequent occupations held by individuals in the four occupational PA trajectory groups at each decade included in the trajectory model. Briefly, the most common occupation was primary education teachers in the stable low group; salespersons (retail and other) in the stable intermediate group; nursing and care assistants in the stable high group; and varied between secretaries (early and late in life) and nursing and care assistants (mid-life) in the increasing then decreasing group. Table S6 shows examples of individual occupational histories in each occupational PA trajectory group. On average, each participant contributed 10.3 occupational data points (range 2–21) to the trajectory model. Age strongly influenced the number of data points contributed, with study participants aged 70–74 years at HUNT4 70+ (n = 2929) contributing a mean of 14.0 data points (range 2–21) and participants aged 95+ at HUNT4 70+ (n = 79) contributing a mean of 2.6 data points (range 2–3). The trajectory and the individual occupational PA plots (Fig. 2 and Figure S2) illustrate stability of occupational PA level from 32 to 65 years of age among the participants.

There were no interactions between trajectories of occupational PA and sex (*p* for interaction = 0.65), age (*p* for interaction = 0.25), or education (*p* for interaction = 0.67) on risk of dementia or MCI. In the age- and sex-adjusted model (Model 1), individuals with *stable high* occupational PA trajectories had higher risks of dementia and MCI compared to individuals with a *stable low* occupational PA trajectory. A similar pattern was observed among individuals with *stable intermediate* occupational PA trajectories. Further, participants with an *increasing then decreasing* occupational PA trajectory had a higher risk of MCI, but not dementia, compared to individuals with a *stable low* trajectory (Fig. 3). Adjusting for education (Model 2) attenuated the results; notably, participants with a *stable intermediate* occupational PA trajectory still had a heightened risk of MCI, but no longer had a higher risk of dementia, whereas participants with a *stable high* occupational PA trajectory continued to demonstrate a heightened risk of dementia and MCI, compared to a *stable low* occupational PA trajectory. Individuals with an *increasing then decreasing* occupational PA trajectory no longer had a higher risk of MCI compared to the *stable low* occupational PA trajectory (Fig. 3). Adjustment for mean annual income at age 45–55 (Model 3) further attenuated the results, particularly the associations with dementia (Fig. 3). Further adjustments for marital status and health and lifestyle-related variables (Model 4) did not appreciably attenuate the associations (Fig. 3), and the *stable high* occupational PA trajectory group continued to exhibit a heightened dementia and MCI risk, whereas the *stable intermediate* trajectory group still had a higher MCI risk compared to the *stable low* trajectory group in the fully adjusted model (Model 4). According to the fully adjusted estimates presented in Fig. 3, when compared to the individuals with a *stable low* trajectory, participants with a *stable high* trajectory had a RRR for dementia of 1.34 (95% CI 1.04–1.73) and MCI of 1.80 (1.54–2.11), whereas participants with a *stable intermediate* trajectory had a RRR for MCI of 1.36 (1.15–1.61). While not statistically significant, participants with *increasing then decreasing* occupational PA had a RRR for dementia of 0.76 (0.53–1.11) and of MCI of 1.18 (0.96–1.46) when compared to the *stable low* PA group.

### Sensitivity analysis

The estimates from the complete case analysis (n = 5940) without IPW did not differ appreciably from the results from the multiple imputed and IPW weighted analyses, although the associations between trajectories of occupational PA and dementia risk had wider 95% CIs and were no longer statistically significant after adjustment for age, sex, education and mean annual income at ages 45–55 (Table S7, Appendix).

## Discussion

Our findings extend those of prior studies,^5^, ^6^, ^7^ demonstrating that registry-based high occupational PA during adulthood relates to a sigificantly greater risk of cognitive impairment later in life. The rich registry-based data on occupational histories at ages 33–65 years together with the thorough clinical assessments of dementia and MCI at ages 70+ years in our study add to the existing literature by providing a life-course perspective on the association between occupational characteristics and later-life cognitive health. Compared to individuals with a stable low occupational PA trajectory, we found that those with a stable high occupational PA trajectory had an increased risk of both dementia and MCI, whereas those with a stable intermediate occupational PA trajectory had an increased risk of MCI. These findings persisted even after adjustment for education, income, marital status, health, and lifestyle-related factors. Individuals whose occupational PA increased and then decreased during midlife did not differ significantly from individuals with a stable low occupational PA trajectory in terms of dementia or MCI risk in adjusted models.

There are several plausible explanations for the observed associations between occupational PA and dementia and MCI. Higher occupational physical demands in later adulthood have previously been linked to smaller hippocampal volume and poorer memory performance.^38^ Similarly, individuals working in physically hazardous jobs^39^ or with high job demands (psychological or physical) combined with low job control^40^ have been found to perform poorer on cognitive tests in later age. This may suggest a detrimental effect of high occupational physical demands on brain health and cognitive function in older ages, which could reflect on risk of later-life cognitive impairment.

Individuals working in physically-demanding professions may differ in terms of genetics, socioeconomic factors and environmental exposures, and confounding due to unobserved variation in these is possible. For instance, people with high occupational PA may have had lower early-life cognitive abilities,^41^^,^^42^ which in turn may influence their school and job market opportunities, which regardless of occupational PA are related to greater risk of dementia and MCI. This suggests that the association between occupational PA and late-life cognitive impairment could be confounded by differences in socioeconomic status. Such a confounding effect was observed in a study on occupational PA and longevity in men, where the association between high occupational PA and increased mortality in men was reversed after adjustment for health, lifestyle, and socioeconomic factors, with estimates suggesting that men in occupations characterized by higher PA levels actually had longer life expectancies than men in the sedentary occupations.^13^ We observed a substantial attenuation of the association between occupational PA trajectories and cognitive impairment, particularly dementia, after adjustment for registry-based education and income. However, our estimates still revealed a detrimental effect of higher occupational PA on risk of dementia and MCI even after adjustment for education, income, marital status, and lifestyle and health-related factors, indicating that differences in socioeconomic status and other potential confounders do not fully explain the increased risk of cognitive impairment in individuals with a history of high occupational PA.

Greater physical demands, lack of recuperation, and resulting exhaustion could imply more somatic ‘wear and tear’ and shorter recovery periods, which could collectively worsen cognition.^43^^,^^44^ Common occupations among those with intermediate or high occupational PA trajectories in our study were salespersons (retail and other), nursing and care assistants, and crop farmers and animal producers. These jobs are often characterized by a lack of autonomy, prolonged standing, hard work, rigid working hours, stress, a higher risk of burnout, and sometimes low socioeconomic status and inconvenient working days.^8^^,^^22^^,^^45^, ^46^, ^47^, ^48^, ^49^, ^50^ Occupations with high physical demands may also be associated with a greater risk of hearing loss and exposure to pollution, which may adversely affect cognition.^51^ In contrast, low PA occupations may often represent work situations with more flexible working hours^52^^,^^53^ allowing for breaks, recovery, and organizing work according to workers' preferences. Moreover, many low PA jobs, such as engineering, administration, and teaching, may be more cognitively stimulating, which could contribute to more favorable cognitive development throughout the course of a person's life.^54^^,^^55^ Hence, occupational characteristics beyond occupational PA may be important contributors to the observed associations between higher levels of occupational PA and risk of cognitive impairment in later life and will need to be further assessed in future studies.

In line with the PA paradox demonstrating opposing effects of leisure-time PA and occupational PA on outcomes such as cardiovascular disease and mortality,^8^, ^9^, ^10^, ^11^, ^12^ there is extensive evidence from observational and experimental studies on the benefits of leisure-time PA for brain health.^56^^,^^57^ However, a recent randomized-controlled exercise trial in older adults aged 70–77 years at baseline showed that high intensity interval training was associated with hippocampal atrophy and adversely affected the composition of neurochemicals in the hippocampus compared to older adults performing at least 30 minutes of moderate PA at least five days/week.^58^^,^^59^ Together with our findings, this may suggest that hard physical exertion in later adulthood, either at work or during leisure-time, may negatively influence brain and cognitive health. However, these inconsistencies warrant further investigations to better understand the impact of the *intensity* of PA, both at work and during leisure-time, on the brain in older ages.

### Strengths and limitations

The present study has several important strengths. We used a large, population-based cohort with clinically-based diagnoses of dementia and MCI, individually linked with rich Norwegian prospective registry data going back to 1960, and incorporating longitudinal information on socioeconomic, health, and admistrative data. These data enabled the construction of occupational PA trajectories to help elucidate how variation in occupational histories is associated with the risk of cognitive impairment. Further, we had available data on non-participants, which allowed us to weight the analyses for non-participation using IPW.

As with most other studies, ours also has a few limitations. Although our use of registry data linked with O^∗^NET is less vulnerable to recall bias than individual self-reported evaluations of occupational physical activity, the availability and quality of data in national registries tend to improve with time. The older individuals in our study had fewer data points as data availability is greater in more recent periods. Although fewer observations suggest lower estimation precision, point estimates are not affected when we drop those aged 90 or older from the analyses.

Further, our use of the 2003 version of O^∗^NET means that we may have missed information on how physically demanding different occupations were in earlier years, particularly in the years 1960, 1970, and 1980. In other words, our analyses did not account for temporal changes in work tasks within the same occupational category.^60^ We also did not have early-life cognitive data, which would have been useful to further assess the influence of early-life cognition on occupational opportunities and later-life cognition. In addition, MCI prevalence was high (35.3%) in HUNT4 70+, possibly due to the 1.0 SD threshold.^27^ Applying a stricter threshold for MCI could have yielded different results. Furthermore, we did not account for competing risk due to death, as our study participants had to survive and participate in HUNT4 70+. If mortality was higher among those with high occupational PA trajectories compared to those with lower occupational PA trajectories,^9^^,^^11^ the competing risk of death would attenuate the associations and hence make our estimates edge on the conservative side. Also, although our study includes comprehensive registry-based data, several of the covariates used in the analysis were based on self-report. This may have led to misclassification due to recall and/or social desirability bias. Hence, there may be variation in the health status of the participants that was not adequately captured by our approach. Residual confounding might play a role, as in most observational studies, and this could occur due to measurement error, reporting biases, crude measures, or the lack of important confounders. We focused on inclusion of confounders as identified by the Lancet commission on dementia prevention.^51^ However, the confounders used in this study could be represented by other measures; for instance, obesity could have been based on waist-to-hip ratio rather than BMI. Furthermore, there are additional risk factors not included by the Lancet commission, such as diet which could play a role for MCI and dementia risk, and might be connected to occupational PA.^61^ Thus, we acknowledge that unobserved factors not accounted for in the analyses may hypothetically bias our estimates. Most past studies do not mutually control for occupational PA and leisure-time PA in their analyses, which is a key strength of the current paper. Our study also gives importance to the role of volume or dose of PA–beneficial effects of leisure activities. While short bouts of moderate to vigorous PA can lower cardiovascular risk factors such as heart rate and blood pressure and improve cardiorespiratory fitness, long durations of high-level PA (common in physically-demanding work) may have the opposite (detrimental) effects on CVD risk factors. Explicit consideration of potential overadjustment by covariates such as CVD that are being considered as both confounders and mediators may have influenced the estimates. However, Model 4, which included CVD (Table S7) is almost identical to the models without this variable (Models 2 and 3), which leads us to believe CVD is not central in this study. Our full model, which includes income, may have underestimated the total effects of occupational PA, since adjustment for mediators removes the indirect paths working via these mediators.

### Conclusion

Results from this registry-based study underline the importance of occupational histories for cognitive health in later life. Our results particularly underscore the need to follow up individuals with high lifetime occupational PA, as they appear to have a greater risk of developing dementia.

While leisure-time PA can lower cardiovascular risk factors such as heart rate and blood pressure, as well as lower the risk of diabetes, long durations of occupational PA may have the opposite (detrimental) effects on cardiovascular disease, MCI and dementia risk. Our study and those of others undescore the need to find technologically-based solutions and ways to reduce high occupational PA across the working life, for instance, through greater work flexibility, job rotation, forced pauses and mandatory recovery periods, as well as possibly imposing a reduction of weekly or lifetime work hours. Future research should assess how occupational PA and interventions to reduce occupational PA or technological changes leading to altered occupational PA, in combination with other occupational characteristics, relate to dementia and MCI risk in older ages to further our understanding of the association between occupational histories and cognitive impairment.

## Contributors

EZ: responsible for the original and final draft of the manuscript, data curation and formal analyses, worked with methodology, contributed to the conceptualization, drafting, and reviewing of the final manuscript.

BB: data curation and formal analyses, worked with methodology, contributed to the conceptualization, drafting, and reviewing of the final manuscript.

BHS: project administrator, data curation and formal analyses, acquired funding, worked with methodology, contributed to the conceptualization, drafting, and reviewing of the final manuscript.

AJ: acquired funding, contributed to the conceptualization, drafting, and reviewing of the final manuscript.

BLE: contributed to the conceptualization, drafting, and reviewing of the final manuscript.

CB: acquired funding, contributed to the conceptualization, drafting, and reviewing of the final manuscript.

GS: acquired funding, contributed to the conceptualization, drafting, and reviewing of the final manuscript.

HPK: acquired funding, contributed to the conceptualization, drafting, and reviewing of the final manuscript.

JRH: acquired funding, contributed to the conceptualization, drafting, and reviewing of the final manuscript.

JW: contributed to the conceptualization, drafting, and reviewing of the final manuscript.

SET: acquired funding, contributed to the conceptualization, drafting, and reviewing of the final manuscript.

SK: contributed to the conceptualization, drafting, and reviewing of the final manuscript.

TM: contributed to the conceptualization, drafting, and reviewing of the final manuscript.

THE: contributed to the conceptualization, drafting, and reviewing of the final manuscript.

YS: acquired funding, contributed to the conceptualization, drafting, and reviewing of the final manuscript.

AKH: project administrator, acquired funding, worked with methodology, contributed to the conceptualization, drafting, and reviewing of the final manuscript.

VS: project leader, contributed significantly to the original draft of the manuscript, acquired funding, worked with methodology, contributed to the conceptualization, drafting, and reviewing of the final manuscript.

## Declaration of sources of funding

This work was supported by the 10.13039/100000002National Institutes of Health (grant number R01AG069109-01). It was also partly supported by the Research Council of Norway through its Centers of Excellence funding scheme (project numbers 2262700, 296297, 288083). The funding agencies played no role in the design, execution, analysis or interpretation of data, or the writing of this manuscript.

## Data sharing statement

The data used for this study were derived from The Trøndelag Health Study (HUNT), https://www.ntnu.edu/hunt. Any research group with a Principal Investigator affiliated with a Norwegian research institute can apply for access to analyze HUNT data. This means that research groups from non-Norwegian countries must find a collaboration partner in Norway to be able to use HUNT material. Each project needs to be approved by the HUNT Data Access Committee (DAC), Regional Committee for Medical and Health Research Ethics, and in some cases also the Data Inspectorate. Due to participant confidentiality, participant data are not publicly available.

## Ethics approval

This study was approved by the HUNT study board of directors and the Regional Committee for Medical and Health Research Ethics. All participants in the HUNT Study gave a written informed consent upon participation.

## Declaration of interests

The authors declare that the research was conducted in the absence of any commercial or financial relationships that could be construed as a potential conflict of interest.

## References

[1] Burzynska A.Z., Jiao Y., Ganster D.C. (2018). Adult-life occupational exposures: enriched environment or a stressor for the aging brain?. Work Aging Retire.

[2] Zotcheva E., Bergh S., Selbaek G. (2018). Midlife physical activity, psychological distress, and dementia risk: the HUNT study. J Alzheimers Dis.

[3] Ogino E., Manly J.J., Schupf N., Mayeux R., Gu Y. (2019). Current and past leisure time physical activity in relation to risk of Alzheimer's disease in older adults. Alzheimer's Dement.

[4] Tolppanen A.M., Solomon A., Kulmala J. (2015). Leisure-time physical activity from mid- to late life, body mass index, and risk of dementia. Alzheimers Dement.

[5] Nabe-Nielsen K., Holtermann A., Gyntelberg F. (2021). The effect of occupational physical activity on dementia: results from the Copenhagen Male Study. Scand J Med Sci Sports.

[6] Rovio S., Kareholt I., Viitanen M. (2007). Work-related physical activity and the risk of dementia and Alzheimer's disease. Int J Geriatr Psychiatry.

[7] Smyth K.A., Fritsch T., Cook T.B., McClendon M.J., Santillan C.E., Friedland R.P. (2004). Worker functions and traits associated with occupations and the development of AD. Neurology.

[8] Krause N., Brand R.J., Arah O.A., Kauhanen J. (2015). Occupational physical activity and 20-year incidence of acute myocardial infarction: results from the kuopio ischemic heart disease risk factor study. Scand J Work Environ Health.

[9] Holtermann A., Mortensen O.S., Burr H., Søgaard K., Gyntelberg F., Suadicani P. (2010). Physical demands at work, physical fitness, and 30-year ischaemic heart disease and all-cause mortality in the Copenhagen Male Study. Scand J Work Environ Health.

[10] Johansson M.S., Holtermann A., Marott J.L. (2022). The physical activity health paradox and risk factors for cardiovascular disease: a cross-sectional compositional data analysis in the Copenhagen City Heart Study. PLoS One.

[11] Coenen P., Huysmans M.A., Holtermann A. (2018). Do highly physically active workers die early? A systematic review with meta-analysis of data from 193,696 participants. Br J Sports Med.

[12] Holtermann A., Hansen J.V., Burr H., Søgaard K., Sjøgaard G. (2012). The health paradox of occupational and leisure-time physical activity. Br J Sports Med.

[13] Dalene K.E., Tarp J., Selmer R.M. (2021). Occupational physical activity and longevity in working men and women in Norway: a prospective cohort study. Lancet Public Health.

[14] World Health Organization (2020).

[15] Quinn T.D. (2020). https://www.proquest.com/openview/c0933fab489b69594e03cbd410977219/1?pq-origsite=gscholar&cbl=18750&diss=y.

[16] Ferrari P., Friedenreich C., Matthews C.E. (2007). The role of measurement error in estimating levels of physical activity. Am J Epidemiol.

[17] Koolhaas C.M., van Rooij F.J., Cepeda M., Tiemeier H., Franco O.H., Schoufour J.D. (2018). Physical activity derived from questionnaires and wrist-worn accelerometers: comparability and the role of demographic, lifestyle, and health factors among a population-based sample of older adults. Clin Epidemiol.

[18] Kowalski K., Rhodes R., Naylor P.J., Tuokko H., MacDonald S. (2012). Direct and indirect measurement of physical activity in older adults: a systematic review of the literature. Int J Behav Nutr Phys Act.

[19] Dubois B., Hampel H., Feldman H.H. (2016). Preclinical Alzheimer's disease: definition, natural history, and diagnostic criteria. Alzheimers Dement.

[20] Statistics Norway (2016). https://www.ssb.no/en/klass/klassifikasjoner/145/.

[21] O∗NET OnLine Work activities & performing general physical activities. http://www.onetonline.org/find/descriptor/result/4.A.3.a.1.

[22] Smith P., Ma H., Glazier R.H., Gilbert-Ouimet M., Mustard C. (2018). The relationship between occupational standing and sitting and incident heart disease over a 12-year period in Ontario, Canada. Am J Epidemiol.

[23] NAV (2022). https://www.nav.no/no/nav-og-samfunn/statistikk/pensjon-statistikk/alderspensjon.

[24] Vassenden K. (1987).

[25] Statistics Norway (2002). https://www.ssb.no/en/klass/klassifikasjoner/6/versjon/31/koder.

[26] Statistics Norway (2001).

[27] Gjøra L., Strand B.H., Bergh S. (2021). Current and future prevalence estimates of mild cognitive impairment, dementia, and its subtypes in a population-based sample of people 70 Years and older in Norway: the HUNT study. J Alzheimers Dis.

[28] Guha M. (2014). Diagnostic and statistical manual of mental disorders: DSM-5 (5th edition). Ref Rev.

[29] Mitchell A.J., Shiri-Feshki M. (2009). Rate of progression of mild cognitive impairment to dementia–meta-analysis of 41 robust inception cohort studies. Acta Psychiatr Scand.

[30] Westhoff L., Bukodi E., Goldthorpe J.H. (2022). Social class and age-earnings trajectories in 14 European countries. Res Soc Stratif Mobil.

[31] World Health Organization (2023). https://www.who.int/health-topics/obesity#tab=tab_1.

[32] Elsawy B., Higgins K.E. (2010). Physical activity guidelines for older adults. Am Fam Physician.

[33] Jones B.L., Nagin D.S. (2013). A note on a Stata plugin for estimating group-based trajectory models. Socio Methods Res.

[34] Nagin D.S. (2005).

[35] Nagin D.S., Odgers C.L. (2010). Group-based trajectory modeling in clinical research. Annu Rev Clin Psychol.

[36] Shiri-Feshki M. (2009).

[37] Shinan-Altman S., Werner P. (2019). Subjective age and its correlates among middle-aged and older adults. Int J Aging Hum Dev.

[38] Burzynska A.Z., Ganster D.C., Fanning J. (2020). Occupational physical stress is negatively associated with hippocampal volume and memory in older adults. Front Hum Neurosci.

[39] Gow A.J., Avlund K., Mortensen E.L. (2014). Occupational characteristics and cognitive aging in the Glostrup 1914 Cohort. J Gerontol B Psychol Sci Soc Sci.

[40] Dong L., Eaton W.W., Spira A.P., Agnew J., Surkan P.J., Mojtabai R. (2018). Job strain and cognitive change: the Baltimore Epidemiologic Catchment Area follow-up study. Occup Environ Med.

[41] Cheng H., Furnham A. (2012). Childhood cognitive ability, education, and personality traits predict attainment in adult occupational prestige over 17 years. J Vocat Behav.

[42] Gottfredson L.S. (1997). Why g matters: the complexity of everyday life. Intelligence.

[43] Chaudhuri A., Behan P.O. (2004). Fatigue in neurological disorders. Lancet.

[44] Ellbin S., Jonsdottir I.H., Eckerström C., Eckerström M. (2021). Self-reported cognitive impairment and daily life functioning 7–12 years after seeking care for stress-related exhaustion. Scand J Psychol.

[45] Gelsema T.I., Van Der Doef M., Maes S., Janssen M., Akerboom S., Verhoeven C. (2006). A longitudinal study of job stress in the nursing profession: causes and consequences. J Nurs Manag.

[46] Salvagioni D.A.J., Melanda F.N., Mesas A.E., González A.D., Gabani F.L., Andrade S.M. (2017). Physical, psychological and occupational consequences of job burnout: a systematic review of prospective studies. PLoS One.

[47] De Jonge J., Dormann C. (2017).

[48] Hartvigsen J., Lings S., Leboeuf-Yde C., Bakketeig L. (2004). Psychosocial factors at work in relation to low back pain and consequences of low back pain; a systematic, critical review of prospective cohort studies. Occup Environ Med.

[49] Krause N., Brand R.J., Kaplan G.A. (2007). Occupational physical activity, energy expenditure and 11-year progression of carotid atherosclerosis. Scand J Work Environ Health.

[50] Krause N., Arah O.A., Kauhanen J. (2017). Physical activity and 22-year all-cause and coronary heart disease mortality. Am J Ind Med.

[51] Livingston G., Huntley J., Sommerlad A. (2020). Dementia prevention, intervention, and care: 2020 report of the Lancet Commission. Lancet.

[52] Backhaus N. (2022). Working time control and variability in europe revisited: correlations with health, sleep, and well-being. Int J Environ Res Publ Health.

[53] Magda I., Lipowska K. (2022).

[54] Wilson R.S., De Leon C.F.M., Barnes L.L. (2002). Participation in cognitively stimulating activities and risk of incident Alzheimer disease. JAMA.

[55] Hertzog C., Kramer A.F., Wilson R.S., Lindenberger U. (2008). Enrichment effects on adult cognitive development. Psychol Sci Publ Interest.

[56] Firth J., Stubbs B., Vancampfort D. (2018). Effect of aerobic exercise on hippocampal volume in humans: a systematic review and meta-analysis. Neuroimage.

[57] Xu W., Wang H.F., Wan Y., Tan C.C., Yu J.T., Tan L. (2017). Leisure time physical activity and dementia risk: a dose-response meta-analysis of prospective studies. BMJ Open.

[58] Pani J., Reitlo L.S., Evensmoen H.R. (2021). Effect of 5 Years of exercise intervention at different intensities on brain structure in older adults from the general population: a generation 100 substudy. Clin Interv Aging.

[59] Reitlo L.S., Mihailovic J.M., Stensvold D., Wisløff U., Hyder F., Håberg A.K. (2023). Hippocampal neurochemicals are associated with exercise group and intensity, psychological health, and general cognition in older adults. Geroscience.

[60] Hanvold T.N., Sterud T., Kristensen P., Mehlum I.S. (2019). Mechanical and psychosocial work exposures. Scand J Work Environ Health.

[61] Charreire H., Kesse-Guyot E., Bertrais S. (2011). Associations between dietary patterns, physical activity (leisure-time and occupational) and television viewing in middle-aged French adults. Br J Nutr.

