# Supplementary material for: Trajectories of occupational physical activity and risk of later-life mild cognitive impairment and dementia: the HUNT4 70+ study
Source: Lancet Reg Health Eur. 2023 Aug 29;34:100721. doi: 10.1016/j.lanepe.2023.100721 (PMC10625024; doi:10.1016/j.lanepe.2023.100721)
Supplement: Appendix [file mmc1.docx]

Appendix for **Trajectories of occupational physical activity and risk of later-life mild cognitive impairment and dementia: The HUNT4 70+ Study**

**Contents**

[Supplementary methods 2](#_Toc137642488)

[Figure S1. 2](#_Toc137642489)

[Group-based trajectory modeling 2](#_Toc137642490)

[Table S1. 3](#_Toc137642491)

[Table S2. 3](#_Toc137642492)

[Table S3. 4](#_Toc137642493)

[Figure S2. 5](#_Toc137642494)

[Inverse probability-weighting and multiple imputation procedure 5](#_Toc137642495)

[Table S4 6](#_Toc137642496)

[Figure S3. 7](#_Toc137642497)

[Table S5. 8](#_Toc137642498)

[Table S6. 10](#_Toc137642499)

[Table S7. 11](#_Toc137642500)

# Supplementary methods


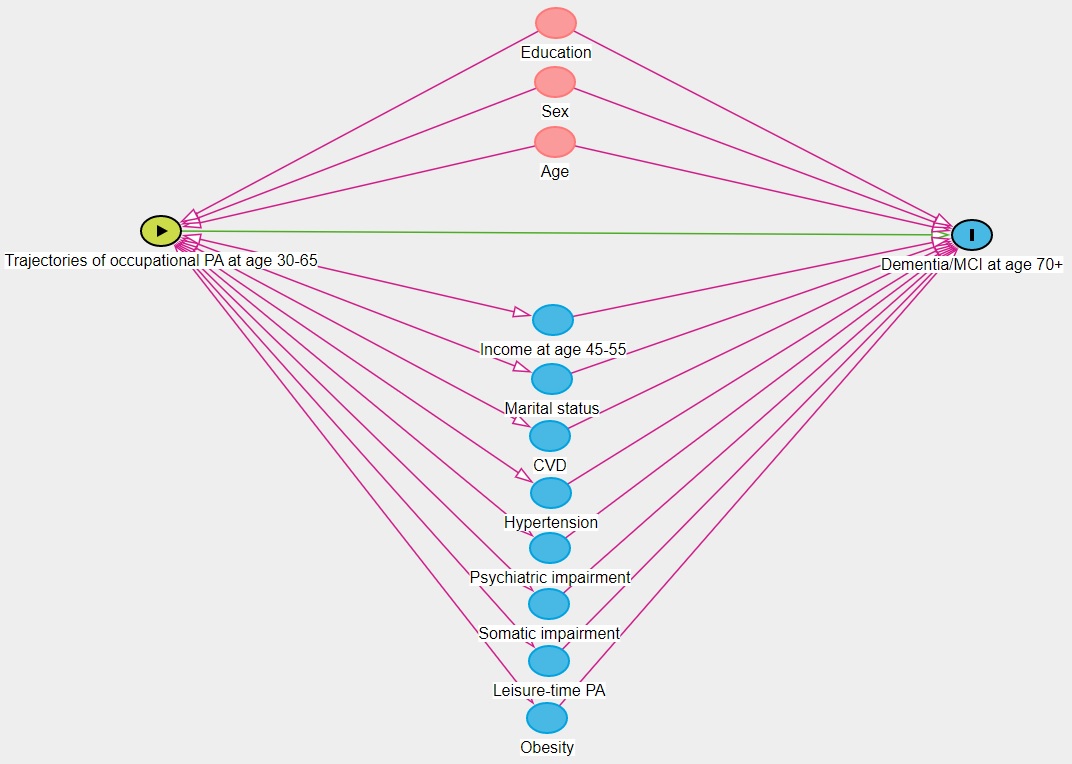


Figure S1. Directed acyclic graph (DAG) of the association between trajectories of occupational PA (exposure) and dementia/MCI at age 70+ (outcome). Pink lines indicate biasing paths, whereas green lines indicate causal paths. Pink circles indicate potential confounders, whereas blue circles indicate potential mediators and/or confounders.

# Group-based trajectory modeling

We used the conventional mean posterior probability of 0.70 as a cut-off to indicate that each trajectory group contained participants with similar occupational PA trajectories at ages 33-65 (25, 26). To ensure sufficient statistical power in the statistical analyses of trajectories of occupational PA and dementia/MCI, a frequency of ≥5% was used for the trajectory groups. Finally, we chose the number and shape of the trajectory groups that reflected the data in a comprehensible and analytically tractable manner. Based on the standard criteria a trajectory model with 5 groups (polynomials 1 2 2 2 2) was found to have the best goodness-of-fit (Supplemental Table 1 and 2). However, one of the groups in this trajectory model was below the preferred frequency (4.9%). When testing other 5-group trajectory models, we experienced issues related to groups being too homogeneous, decreasing interpretability. Hence, to increase interpretability and statistical power, we selected the trajectory model with 4 groups that had the best fit to data and classification accuracy (polynomials 2 2 2 2, Supplemental Table 2 and 3).

| Table S1. Characteristics for trajectory modeling. | | | | | | | |
| --- | --- | --- | --- | --- | --- | --- | --- |
| No of groups | Polynomials | LL | BIC | AIC | 2*ΔBIC | Group size (range) | Mean PP (range) |
| 2 | 22 | -74509.07 | -74553.80 | -74517.07 | . | 38.2-61.8% | 0.987-0.989 |
| 3 | 222 | -66895.94 | -66949.06 | -66907.94 | 15209.48 | 27.9-40.3% | 0.911-0.984 |
| 4 | 2222 | -62780.35 | -62851.19 | -62796.35 | 8195.74 | 9.7-35.5% | 0.885-0.985 |
| 5 | 22222 | -61871.27 | -61959.82 | -61891.27 | 1782.74 | 9.5-30.1% | 0.783-0.986 |
| 6 | 222222 | -60387.31 | -60521.51 | -60411.31 | 1438.31 | 4.6-29.8% | 0.823-0.981 |
| 7 | 2222222 | -59053.49 | -59210.05 | -59081.49 | 2622.92 | 4.5-26.0% | 0.836-0.972 |
| LL: log likelihood, BIC: Bayesian Information Criterion, AIC: Akaike Informational Criteria, PP: posterior probability. | | | | | | | |

| Table S2. Comparison of four- and five-group trajectory model shapes of occupational physical activity, sorted by descending Bayesian Information Criterion (BIC). Bold font indicates the four- and five-group trajectory models with the best fit to data. | | |
| --- | --- | --- |
| Groups | Polynomials | BIC |
| 1 | 1 | -101480.24 |
| 1 | 2 | -101422.55 |
| 2 | 11 | -74242.034 |
| 2 | 21 | -74216.357 |
| 2 | 12 | -74085.64 |
| 2 | 22 | -74076.172 |
| 3 | 111 | -67145.655 |
| 3 | 211 | -67143.097 |
| 3 | 112 | -67094.22 |
| 3 | 212 | -67091.611 |
| 3 | 221 | -67091.611 |
| 3 | 121 | -66519.634 |
| 3 | 122 | -66502.651 |
| 3 | 222 | -66485.238 |
| 4 | 1211 | -65507.368 |
| 4 | 1111 | -65163.068 |
| 4 | 2111 | -65150.888 |
| 4 | 1121 | -65146.458 |
| 4 | 2112 | -65132.213 |
| 4 | 2121 | -65132.213 |
| 4 | 1112 | -65120.069 |
| 5 | 11111 | -64277.229 |
| 5 | 11112 | -63687.426 |
| 5 | 11121 | -63687.426 |
| 5 | 12122 | -63637.384 |
| 4 | 1122 | -62582.481 |
| 4 | 2211 | -62579.404 |
| 4 | 1212 | -62462.227 |
| 4 | 1221 | -62462.227 |
| 4 | 2212 | -62435.241 |
| 4 | 2221 | -62435.241 |
| 4 | 1222 | -62426.29 |
| 4 | 2122 | -62426.29 |
| **4** | **2222** | **-62398.951** |
| 5 | 12121 | -61758.119 |
| 5 | 21211 | -61758.119 |
| 5 | 12111 | -61756.874 |
| 5 | 11212 | -61732.182 |
| 5 | 22121 | -61730.773 |
| 5 | 22111 | -61728.84 |
| 5 | 21222 | -61697.205 |
| 5 | 22122 | -61697.205 |
| 5 | 21221 | -61559.901 |
| 5 | 21212 | -61541.485 |
| 5 | 22112 | -61541.485 |
| 5 | 22221 | -61539.887 |
| 5 | 22222 | -61520.49 |
| 5 | 11211 | -61385.289 |
| 5 | 21111 | -61385.289 |
| 5 | 21112 | -61368.086 |
| 5 | 11122 | -61364.026 |
| 5 | 12112 | -61364.026 |
| 5 | 11221 | -61296.6 |
| 5 | 12212 | -61285.076 |
| 5 | 11222 | -61266.813 |
| 5 | 12221 | -61266.813 |
| 5 | 21122 | -61266.813 |
| 5 | 12211 | -60963.739 |
| 5 | 21121 | -60963.739 |
| 5 | 22211 | -60951.643 |
| 5 | 22212 | -60948.993 |
| **5** | **12222** | **-60928.676** |

| Table S3. Posterior probabilities and odds of correct classification (OCC) for chosen trajectory model (polynomials 2 2 2 2), n=7,005. | | | | |  |
| --- | --- | --- | --- | --- | --- |
|  | N (%) | Mean posterior probability (SD) | Min. posterior probability | Max. posterior probability | OCC |
| Stable low | 2,162 (30.9%) | 0.99 (0.06) | 0.37 | 1 | 155.19 |
| Increasing then decreasing | 625 (8.9%) | 0.92 (0.15) | 0.40 | 1 | 106.77 |
| Stable intermediate | 1,755 (25.1%) | 0.89 (0.17) | 0.37 | 1 | 24.32 |
| Stable high | 2,463 (35.2%) | 0.95 (0.11) | 0.40 | 1 | 36.29 |

Figure S2. Plot of 100 randomly drawn individual trajectories of standardized occupational PA index by age, stratified by occupational PA trajectory group.

# Inverse probability-weighting and multiple imputation procedure

Multiple imputation with 20 iterations was used to impute missing values on adjustment variables (27). There were no missing values in our study population for the registry-based variables age, sex, education, trajectories of occupational PA, or income, and these variables were thus used as non-missing variables in the prediction, together with cognitive status assessed at HUNT4 70+. The following numbers were imputed for the study population (n=7,005): hypertension (n=457, 6.5%), CVD (n=452, 6.5%), marital status (n=11, 0.2%), psychiatric illness (n=739, 10.6%), somatic illness (n=464, 6.6%), insufficient leisure-time PA (n=789, 11.3%), and obesity (n=461, 6.6%).

Inverse-probability weighting (IPW) was applied to account for non-response in HUNT4 70+ and correct for bias due to skewed participation concerning age, sex, and education. The probability of participation was calculated by performing a logistic regression model including registry-based data on age, sex, and education available for all invited to HUNT4 70+ (n=19,463, of whom 9,930 participated). We used the inverse of the probability to weigh the results from the multinominal logistic regression.

| Table S4**.** Characteristics of three subgroups of 2018 Nord-Trøndelag population: (1) study sample; (2) HUNT4 70+ participants with insufficient occupational data; and (3) non-participants in HUNT4 70+. | | | |
| --- | --- | --- | --- |
|  | (1)  Study sample  (n=7005) | (2)  HUNT4 70+ participants with insufficient occupational data  (n=2840) | (3)  Non-participants in HUNT4 from the Nord-Trøndelag population aged 70+, with occupational data  (n=7554) |
| **Registry-based** |  |  |  |
| Age in 2018 | 77.3 (6.6) | 80.3 (6.8) | 78.0 (7.3) |
| Women % | 49.8 (50.0) | 66.1 (47.4) | 46.0 (49.8) |
| Education |  |  |  |
| Primary % | 20.4 (40.3) | 39.4 (48.9) | 32.9 (47.0) |
| Secondary % | 54.0 (49.8) | 53.0 (49.9) | 51.2 (50.0) |
| Tertiary % | 25.6 (43.7) | 7.6 (26.5) | 15.9 (36.6) |
| Married % | 87.6 (33.0) | 84.9 (35.8) | 83.2 (37.4) |
| Retirement age | 65.3 (4.1) | 63.6 (6.2) | 64.8 (4.4) |
| Annual income age 45-55 years, EUR | 53,243 (25,392) | 29,057 (25,180) | 49,735 (23,734) |
| **Occupational PA** |  |  |  |
| Standardized occupational PA index |  |  |  |
| Age 30-39 | -0.02 (0.98) |  | 0.15 (0.95) |
| Age 40-49 | 0.08 (0.98) |  | 0.16 (0.96) |
| Age 50-59 | 0.03 (1.00) |  | 0.16 (0.98) |
| Age 60-69 | -0.05 (1.01) |  | 0.11 (1.00) |
| **HUNT1-HUNT2** |  |  |  |
| Insufficient leisure-time PA % | 43.9 (49.6) | 53.3 (49.9) |  |
| Obesity % | 14.9 (35.6) | 20.7 (40.5) |  |
| Hypertension % | 51.3 (50.0) | 60.1 (49.0) |  |
| CVD % | 3.9 (19.3) | 6.8 (25.1) |  |
| Psychiatric impairment % | 2.5 (15.6) | 6.0 (23.8) |  |
| Somatic impairment % | 8.2 (27.4) | 19.1 (39.3) |  |
| **HUNT4 70+** |  |  |  |
| Dementia % | 12.9 (33.5) | 22.8 (41.9) |  |
| MCI % | 34.4 (47.5) | 36.4 (48.1) |  |
| Note: Numbers in parentheses are standard deviations. Study sample in column (1) is restricted to those with valid cognition data and at least one year with occupational data at ages below and above age 50 years. Sample in column (2) is restricted to those with valid cognition data, and sample in column (3) to those aged 70 years or more in 2018 with at least one year with occupational data at ages below and above age 50 years. Occupational PA index is standardized to have mean zero and standard deviation one in study sample. An overview of the distribution of occupational physical activity index in the study sample and the non-participant group is presented in Figure S3. CVD: cardiovascular disease; EUR: Euro; HUNT: Trøndelag Health Study; HUNT1: 1984-1996; HUNT2: 1995-1997; HUNT4 70+: 2017-2019; MCI: mild cognitive impairment, PA: physical activity. | | | |

Figure S3. Distribution of occupational physical activity (PA) index in study sample and among non-participants in HUNT4 70+ survey.

Note: Density plots are based on data set with person-by-year observations, age range 30 to 65 years. Sample of non-participants restricted to 2018 Nord-Trøndelag population aged 70 or above with occupational data both before and after age 50. Observation count is 71,927 in study sample (unique individuals 7,005) and 69,990 in sample of non-participants (unique individuals 7,554). Mean occupational PA in study sample is 3.06 and standard deviation is 0.93; standardized score used elsewhere in manuscript subtracts 3.06 from the observed occupational PA and divides the difference by 0.93.

| Table S5. Most common occupations (4-digit level) held by participants from the HUNT4 70+ Study (n=7,005) by age and trajectory of occupational physical activity group. | | | | | | | | | | | | |
| --- | --- | --- | --- | --- | --- | --- | --- | --- | --- | --- | --- | --- |
|  | Stable low (n=2,162) | | | Increasing then decreasing (n=625) | | | Stable intermediate (n=1,755) | | | Stable high (n=2,463) | | |
| Age | Occupational code | Occupation | Percent | Occupational code | Occupation | Percent | Occupational code | Occupation | Percent | Occupational code | Occupation | Percent |
| 30s |  |  |  |  |  |  |  |  |  |  |  |  |
|  | 3310 | Primary education teaching associate professionals | 33.8% | 4113 | Secretaries | 18.2% | 5221 | Shop salespersons and other salespersons (retail) | 16.1% | 6130 | Crop and animal producers | 13.3% |
|  | 4113 | Secretaries | 8.8% | 5221 | Shop salespersons and other salespersons (retail) | 7.4% | 6130 | Crop and animal producers | 15.4% | 5132 | Nursing assistants and care assistants | 9.2% |
|  | 3111 | Civil engineering technicians | 4.7% | 1210 | Directors and chief executives | 6.4% | 4113 | Secretaries | 3.9% | 5221 | Shop salespersons and other salespersons (retail) | 6.4% |
| 40s |  |  |  |  |  |  |  |  |  |  |  |  |
|  | 3310 | Primary education teaching associate professionals | 16.0% | 6130 | Crop and animal producers | 6.7% | 5221 | Shop salespersons and other salespersons (retail) | 16.8% | 5132 | Nursing assistants and care assistants | 9.1% |
|  | 4113 | Secretaries | 4.7% | 5132 | Nursing assistants and care assistants | 5.0% | 6130 | Crop and animal producers | 12.3% | 9132 | Helpers and cleaners in offices and other establishments | 5.9% |
|  | 2419 | Other public service administrative professionals | 2.7% | 5131 | Child-care workers | 3.4% | 4113 | Secretaries | 3.6% | 6130 | Crop and animal producers | 5.0% |
| 50s |  |  |  |  |  |  |  |  |  |  |  |  |
|  | 3310 | Primary education teaching associate professionals | 25.6% | 5131 | Child-care workers | 8.8% | 5221 | Shop salespersons and other salespersons (retail) | 26.2% | 5131 | Child-care workers | 11.7% |
|  | 2320 | Secondary education teaching professionals | 6.2% | 5221 | Shop salespersons and other salespersons (retail) | 6.9% | 6130 | Crop and animal producers | 8.6% | 5132 | Nursing assistants and care assistants | 11.4% |
|  | 2419 | Other public service administrative professionals | 5.6% | 5132 | Nursing assistants and care assistants | 6.6% | 7241 | Electricians, electrical and electronic equipment mechanics and fitters | 2.8% | 3231 | Nurses | 7.2% |
| 60s |  |  |  |  |  |  |  |  |  |  |  |  |
|  | 3310 | Primary education teaching associate professionals | 25.1% | 4113 | Secretaries | 9.6% | 5221 | Shop salespersons and other salespersons (retail) | 21.2% | 5132 | Nursing assistants and care assistants | 10.7% |
|  | 2419 | Other public service administrative professionals | 5.4% | 4114 | Clerical officers | 6.1% | 6130 | Crop and animal producers | 5.3% | 5131 | Child-care workers | 8.9% |
|  | 2320 | Secondary education teaching professionals | 4.7% | 2419 | Other public service administrative professionals | 4.3% | 5139 | Personal care and related workers not elsewhere classified | 2.4% | 5139 | Personal care and related workers not elsewhere classified | 8.0% |

| Table S6. Examples of individual occupational histories in the four occupational physical activity trajectory groups, by age. Numbers in brackets are 4-digit occupation codes. | | | | |
| --- | --- | --- | --- | --- |
|  | 30s | 40s | 50s | 60s |
| Stable low |  |  |  |  |
| Person 1 | Primary education teaching associate professional (3310) | Primary education teaching associate professional (3310) | Primary education teaching associate professional (3310) | Primary education teaching associate professional (3310) |
| Person 2 | Psychologist (2545) | Psychologist (2545) | Psychologist (2545) | Psychologist (2545) |
| Person 3 | Primary education teaching associate professional (3310) | Secondary education teaching professional (2320) | Secondary education teaching professional (2320) | Secondary education teaching professional (2320) |
| Person 4 | Production/operations department manager in pubic administration (1227) | Production/operations department manager in pubic administration (1227) | Secondary education teaching professional (2320) | Secondary education teaching professional (2320) |
| Person 5 | Public service administrative professional (2419) | Administrative secreatry/related associate professional (3431) | Administrative secreatry/related associate professional (3431) | Public service administrative associate professional (3449) |
| Increasing then decreasing |  |  |  |  |
| Person 1 | Secretary (4113) | Child-care worker (5131) | Child-care worker (5131) | Secretary (4113) |
| Person 2 | Crop and animal producer (6130) | Crop and animal producer (6130) | Food- and related products machine operator (8279) | Food- and related products machine operator (8279) |
| Person 3 | Civil engineering technician (3111) | Plastic-products machine operator (8224) | Plastic-products machine operator (8224) | Secretary (4113) |
| Person 4 | Secretary (4113) | Production and operations department manager in transport and communications (1225) | Locomotive-engine driver/locomotive-inspector (8311) | Locomotive-engine driver/locomotive-inspector (8311) |
| Person 5 | Stock clerk (4131) | Construction/maintenance laborer (9310) | Construction/maintenance laborer (9310) | Bus/tram driver (8322) |
| Stable intermediate |  |  |  |  |
| Person 1 | Cement/other mineral products machine operator (8214) | Cement/other mineral products machine operator (8214) | Cement/other mineral products machine operator (8214) | Cement/other mineral products machine operator (8214) |
| Person 2 | Social worker (college-trained)/child care officer (3460) | Social worker (college-trained)/child care officer (3460) | Social worker (college-trained)/child care officer (3460) | Production/operations department manager in education, health and social security (1228) |
| Person 3 | Secretary (4113) | Shop salesperson/other salesperson (retail) (5221) | Caretaker (5163) | Shop salesperson/other salesperson (retail) (5221) |
| Person 4 | Shop salesperson/other salesperson (retail) (5221) | Shop salesperson/other salesperson (retail) (5221) | Shop salesperson/other salesperson (retail) (5221) | Shop salesperson/other salesperson (retail) (5221) |
| Person 5 | Electrical line installer/ repairers/cable jointers (7244) | Electricians/electrical and electronic equipment mechanic/fitter (7241) | Electricians/electrical and electronic equipment mechanic/fitter (7241) | Electricians/electrical and electronic equipment mechanic/fitter (7241) |
| Stable high |  |  |  |  |
| Person 1 | Caretaker (5163) | Caretaker (5163) | Caretaker (5163) | Caretaker (5163) |
| Person 2 | Helper/cleaner in office/other establishment (9132) | Nursing assistant/care assistant (5132) | Nursing assistant/care assistant (5132) | Other personal care/related worker (5139) |
| Person 3 | Carpenter/joiner (7125) | Wood-processing-plant operator (8141) | Wood-processing-plant operator (8141) | Carpenter/joiner (7125) |
| Person 4 | Road/construction worker (7126) | Mail carrier/sorting clerk (4142) | Mail carrier/sorting clerk (4142) | Mail carrier/sorting clerk (4142) |
| Person 5 | Motor vehicle mechanic/fitter (7231) | Motor vehicle mechanic/fitter (7231) | Motor vehicle mechanic/fitter (7231) | Motor vehicle mechanic/fitter (7231) |

| Table S7. Comparison of the associations of occupational physical activity (PA) trajectories at ages 33-65 with dementia and MCI risk in complete case analyses without inverse probability weighting (IPW) (n=5,940) and analyses on multiple imputed (MI) data with IPW (n=7,005). | | | | | | | | |
| --- | --- | --- | --- | --- | --- | --- | --- | --- |
|  | Model 1 | | Model 2 | | Model 3 | | Model 4 | |
|  | RRR (95% CI) | | RRR (95% CI) | | RRR (95% CI) | | RRR (95% CI) | |
|  | Complete case | MI and IPW | Complete case | MI and IPW | Complete case | MI and IPW | Complete case | MI and IPW |
| **Dementia** |  |  |  |  |  |  |  |  |
| Stable low | 1.00 (ref.) | 1.00 (ref.) | 1.00 (ref.) | 1.00 (ref.) | 1.00 (ref.) | 1.00 (ref.) | 1.00 (ref.) | 1.00 (ref.) |
| Increasing then decreasing | 1.05 (0.71-1.56) | 1.16 (0.81-1.66) | 0.77 (0.51-1.16) | 0.86 (0.59-1.25) | 0.69 (0.46-1.06) | 0.78 (0.53-1.14) | 0.68 (0.44-1.03) | 0.76 (0.52-1.11) |
| Stable intermediate | 1.70 (1.34-2.16) | 1.74 (1.39-2.18) | 1.17 (0.90-1.54) | 1.21 (0.94-1.56) | 1.03 (0.78-1.36) | 1.05 (0.81-1.37) | 1.02 (0.77-1.35) | 1.04 (0.80-1.36) |
| Stable high | 2.34 (1.87-2.93) | 2.44 (1.97-3.01) | 1.53 (1.18-1.98) | 1.59 (1.25-2.03) | 1.30 (0.99-1.70) | 1.36 (1.05-1.74) | 1.29 (0.98-1.69) | 1.34 (1.04-1.73) |
| **MCI** |  |  |  |  |  |  |  |  |
| Stable low | 1.00 (ref.) | 1.00 (ref.) | 1.00 (ref.) | 1.00 (ref.) | 1.00 (ref.) | 1.00 (ref.) | 1.00 (ref.) | 1.00 (ref.) |
| Increasing then decreasing | 1.41 (1.14-1.75) | 1.36 (1.11-1.66) | 1.28 (1.02-1.60) | 1.21 (0.98-1.49) | 1.27 (1.01-1.60) | 1.20 (0.97-1.49) | 1.25 (1.00-1.58) | 1.18 (0.96-1.46) |
| Stable intermediate | 1.64 (1.40-1.92) | 1.62 (1.40-1.87) | 1.45 (1.22-1.74) | 1.40 (1.19-1.65) | 1.43 (1.19-1.70) | 1.36 (1.16-1.61) | 1.42 (1.19-1.70) | 1.36 (1.15-1.61) |
| Stable high | 2.26 (1.96-2.61) | 2.21 (1.94-2.53) | 2.00 (1.70-2.36) | 1.90 (1.63-2.21) | 1.94 (1.64-2.30) | 1.82 (1.55-2.13) | 1.92 (1.62-2.28) | 1.80 (1.54-2.11) |
| Model 1: age- and sex-adjusted; Model 2: Model 1 + education; Model 3: Model 2 + mean annual income at age 45-55; Model 4: Model 3 + marital status, hypertension, CVD, psychiatric disorders, somatic disorders, insufficient leisure-time PA, and obesity. | | | | | | | | |
